# Mining the transcriptome of target tissues of autoimmune and degenerative pancreatic β-cell and brain diseases to discover therapies

**DOI:** 10.1016/j.isci.2022.105376

**Published:** 2022-10-17

**Authors:** Xiaoyan Yi, Bianca Marmontel de Souza, Toshiaki Sawatani, Florian Szymczak, Lorella Marselli, Piero Marchetti, Miriam Cnop, Decio L. Eizirik

**Affiliations:** 1 ULB Center for Diabetes Research, Medical Faculty, Université Libre de Bruxelles, Brussels 1070, Belgium; 2 Interuniversity Institute of Bioinformatics in Brussels, Université Libre de Bruxelles-Vrije Universiteit Brussel, Brussels 1050, Belgium; 3 Department of Clinical and Experimental Medicine, AOUP Cisanello University Hospital, University of Pisa, Pisa 56126, Italy; 4 Division of Endocrinology, Erasmus Hospital, Université Libre de Bruxelles, Brussels 1070, Belgium; 5 WELBIO, Université Libre de Bruxelles, Brussels 1070, Belgium

**Keywords:** Biological sciences, Immunology, Transcriptomics

## Abstract

Target tissues of autoimmune and degenerative diseases show signals of inflammation. We used publicly available RNA-seq data to study whether pancreatic β-cells in type 1 and type 2 diabetes and neuronal tissue in multiple sclerosis and Alzheimer’s disease share inflammatory gene signatures. We observed concordantly upregulated genes in pairwise diseases, many of them related to signaling by interleukins and interferons. We next mined these signatures to identify therapies that could be re-purposed/shared among the diseases and identified the bromodomain inhibitors as potential perturbagens to revert the transcriptional signatures. We experimentally confirmed in human β-cells that bromodomain inhibitors I-BET151 and GSK046 prevent the deleterious effects of the pro-inflammatory cytokines interleukin-1β and interferon-γ and at least some of the effects of the metabolic stressor palmitate. These results demonstrate that key inflammation-induced molecular mechanisms are shared between β-cells and brain in autoimmune and degenerative diseases and that these signatures can be mined for drug discovery.

## Highlights

• Similar gene transcription signatures in diabetes, multiple sclerosis, and Alzheimer’s
• Inflammatory mechanisms are present in the target tissues of the four diseases
• Common gene expression signatures were mined for the identification of drug targets
• Bromodomain inhibitors decrease islet inflammation in models of types 1 and 2 diabetes

## Graphical abstract

**Figure:**
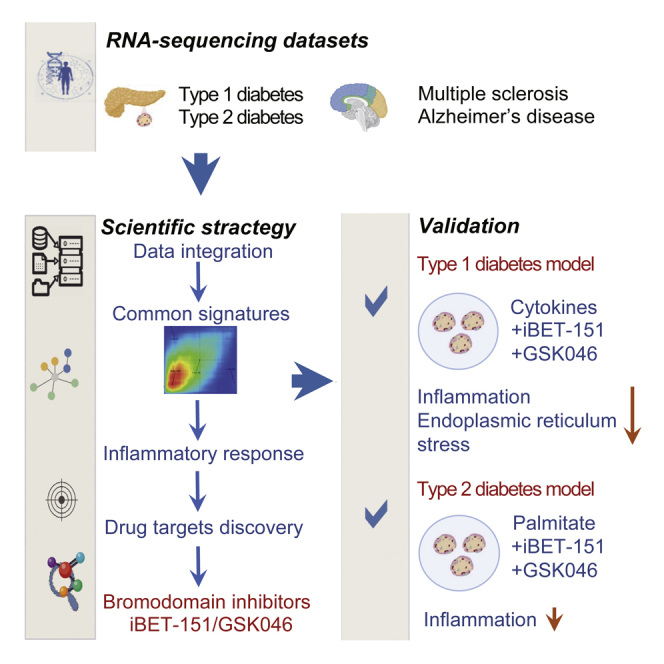

## Introduction

Autoimmune diseases are diseases of “mistaken identity” where the immune system – which is supposed to protect us against infectious diseases and neoplasias – attacks and destroys components of our body. There is no cure for autoimmune diseases and their incidence is increasing worldwide. These conditions – including type 1 diabetes (T1D) and multiple sclerosis (MS) – affect up to 5–8% of the population in different regions.^1^ Although the immune targets of these diseases are distinct, they share several features, including up to 50% common genetic risk loci, chronic local inflammation, and consequently target tissue damage.^1^^,^^2^ Other highly prevalent degenerative diseases, such as type 2 diabetes (T2D) and Alzheimer’s disease (AD), show inflammatory but not autoimmune components.^3^^,^^4^^,^^5^^,^^6^ Despite these common features, autoimmune disorders are traditionally studied independently and with a focus on the immune system rather than on target tissues. There is increasing evidence that the target tissues are not innocent bystanders of the autoimmune attack but participate in a deleterious dialogue with the immune system that contributes to their own demise as shown in a recent study by our group.^7^ This dialogue is supported by changes in the proteome induced by inflammatory mediators that amplify autoimmune responses.^8^ Furthermore, in T1D, several of the risk genes for the disease act at the target tissue level (i.e., pancreatic β-cells), regulating the responses to viral infections,^9^ the dialogue with the immune system and apoptosis.^10^^,^^11^ We hypothesize that key inflammatory mechanisms, potentially shared between T1D, MS, T2D and AD, may induce similar molecular signatures at the target tissue level. Discovering similar (or, in some cases, divergent) signatures may allow the identification of key pathways that could be mined and then, based on the information obtained, targeted for therapy based for instance on the repurposing of drugs already in clinical use for other diseases.

The rationale for selecting these β-cell and brain diseases includes: (1) The striking gene expression similarity between pancreatic β-cells and neurons, including expression of splicing regulators and splice variants^12^^,^^13^; (2) the fact that T1D and MS have several candidate genes in common, and express – at least to some extent – similar upregulated inflammatory pathways at the target tissue levels^7^; and (3) the potential role for inflammation and amyloid deposition in T2D and AD.^5^^,^^6^

We focused on the molecular mechanisms triggered in the target tissues of these diseases, to discover therapies that could reverse commonly perturbed pathways and thus have potential use in the four diseases studied. Our results indicate, at least in part, similar gene expression alterations at the target tissues, many belonging to pathways regulating inflammation. Using Connectivity Map^14^ analyses, we identified potential therapeutic candidates and experimentally validated one of them, bromodomain inhibitors, showing that they protect human β-cells against immune and metabolic stresses of relevance in type 1 and type 2 diabetes, respectively.

## Results

### Data origin and metadata analysis across four diseases

The RNA-seq datasets utilized in the present study were previously generated from fluorescence-activated cell sorting (FACS)-purified pancreatic β-cells from patients affected by T1D,^15^ pancreatic islets from patients affected by T2D,^16^^,^^17^^,^^18^ optical chiasm autopsies for patients affected by MS^19^ and dorsolateral prefrontal cortex autopsies of patients with AD,^20^ and their respective healthy controls (Table 1). We have previously analyzed the T1D- and MS-related data in a comparison against two other autoimmune diseases, rheumatoid arthritis and systemic lupus erythematosus,^7^ and these data were re-analyzed now in comparison against two degenerative diseases with an inflammatory component, namely T2D and AD. Age and sex were obtained from the initial metadata and from our previous study predicting the missing sex information by using the expression of Y chromosome genes (e.g., *SRY* and *PRY2*) and *XIST* (X-inactive specific transcript).^7^

**Table 1:** Overview of the RNA-seq metadata for the four diseases

Disease	Target tissue	Samples (n)		Age (years)		Source	Genes measured
		Patients	Controls	Patients	Controls		
T1D	Pancreatic β-cells	4 (3M/1F)	12 (8M/4F)	20.3 ± 5.6	16.1 ± 5.8	GSE121863	26111
T2D	Pancreatic islets	28 (13F/15M)	183 (77F/106M)	67.8 ± 10.6	65.0 ± 15.7	GSE50244 GSE159984 EGAS00001005535	21038
MS	Optic chiasm	5 (5F)	5 (5F)	56.2	57.6	GSE100297	26718
AD	Prefrontal cortex	122 (95F/27M)	80 (61F/19M)	89.5 ± 3.0	87.8 ± 4.9	Syn21589959	19992

RNA-seq data of target tissues from type 1 diabetes (T1D), type 2 diabetes (T2D), multiple sclerosis (MS) and Alzheimer’s Disease (AD) were gathered from the Gene Expression Omnibus (GEO) portal, European Genome-Phenome Archive (EGA, https://ega-archive.org) and Synapse platform (https://www.synapse.org). M male, F female. Age is displayed as mean ± SD.

The age and sex of the patients reflect the characteristics of the diseases studied, e.g., patients with T2D and AD were older than T1D patients, and females prevailed in MS. Age and sex were well balanced between cases and controls for each individual disease.

The T1D samples consisted of FACS-purified pancreatic β-cells, whereas no purification was done for the other diseases, raising the possibility of significant infiltration by immune cells. To address this, we determined expression of the leukocyte marker CD45 in all samples. There was increased *CD45* expression in T2D and AD, but it remained fairly low, particularly for T2D (Table S1). For comparison, although the mean transcript per million (TPM) for *CD45* in T1D, T2D and their controls ranged from 1 to 15.7, the mean TPM for the β-cell markers in control islet preparations were *INS* (Insulin), 25,568; *FXYD2* (Sodium/potassium-transporting ATPase gamma chain, 219; *GCK*(Glucokinase), 9; *NKX2-2* (Homeobox protein Nkx-2.2), 12; *SYT4* (Synaptotagmin 4), 52; *NEUROD1* (Neurogenic Differentiation 1), 45; *NKX6-1* (Homeobox protein Nkx-6.1), 24; and *MAFB* (MAF BZIP Transcription Factor B), 49. These results indicate that the constitutive cells of the target tissues are the main drivers of transcriptomic alterations.

### Inflammatory genes are upregulated in target tissues of the four diseases

**Figure 1:**
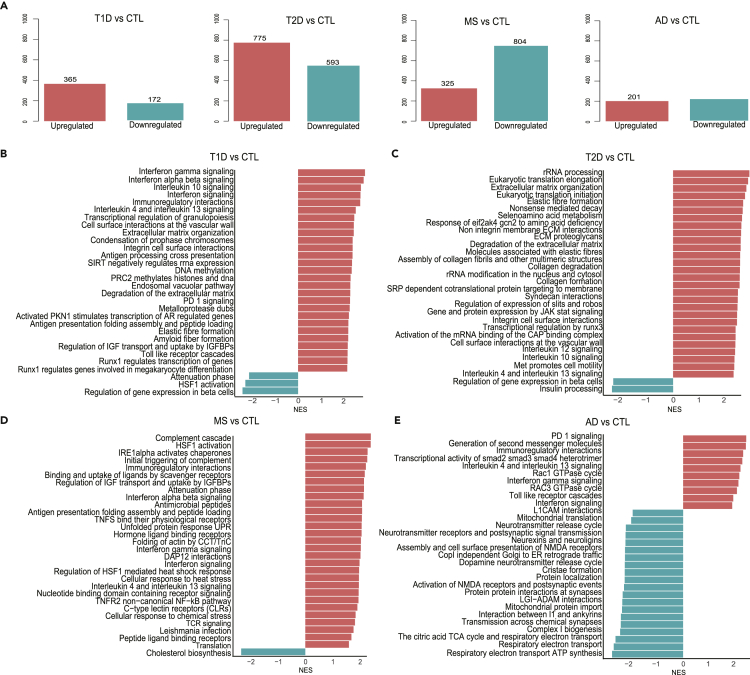
Overview of differentially expressed genes and top 30 enriched Reactome signaling pathways in the target tissues of the four diseases following GSEA analysis (A–D) (A) The number of genes differentially expressed in type 1 diabetes (T1D), type 2 diabetes (T2D), multiple sclerosis (MS), and Alzheimer’s disease (AD). The numbers above the bars represent the genes with an adjusted p-value <0.05. GSEA of T1D (B), T2D (C), MS (D), and AD (E) target tissues based on the Reactome database. Bars in red and blue represent enrichment or inhibition of pathways, respectively. The x-axis shows the normalized enrichment score (NES) of the *fGSEA* analysis, and the y-axis shows enriched pathways with an adjusted p-value <0.05. The full name of pathway “Immunoregulatory interactions” in (B, D and E) is “Immunoregulatory interactions between a lymphoid and a non-lymphoid cell”; “Runx1 regulates transcription of genes” in A is “Runx1 regulates transcription of genes involved in differentiation of HSCS”; “Runx1 regulates genes involved in megakaryocyte differentiation” in A is “Runx1 regulates genes involved in megakaryocyte differentiation and platelet function”; “Gene and protein expression by JAK stat signaling ” in B is “Gene and protein expression by JAK stat signaling after interleukin-2 stimulation”; “Respiratory electron transport ATP synthesis” in D is “Respiratory electron transport ATP synthesis and heat production” (see also Figure S1).

Differential analysis of the modified genes indicated more up-than downregulated genes in T1D and T2D, whereas there were more downregulated genes in MS and AD (Figure 1A). Gene set enrichment analysis (GSEA) based on the Reactome database^21^ showed that interferon (IFN)-γ-regulated pathways – an indicator of adaptive immunity - were augmented in T1D, MS and AD, but not in T2D (Figures 1B–1E). Antigen processing and presentation and IFNα/β signaling pathways were induced in T1D and MS (Figures 1B and 1D). These results were supported by GSEA based on the Kyoto Encyclopedia of Genes and Genomes (KEGG) database,^22^ which also indicated upregulation of antigen presentation in AD (Figures S1A, S1C, and S1D). The “type 1 diabetes” pathway was enriched not only in T1D but also in MS and AD, pointing to resemblance of disease-related genes in target tissues of T1D, MS and AD (Figures S1A, S1C, and S1D). We also found induction of “apoptosis” in T1D, T2D and MS, but not in AD (Figures S1A–S1D). Of interest, “cytokine-cytokine receptor interaction” and other inflammatory components (e.g., “chemokine signaling” and “JAK stat signaling”) were among the top enriched pathways in four diseases (Figure S1), suggesting that these diseases contain similar inflammatory molecular signatures.

Different from the upregulated genes, the enriched pathways for downregulated genes were mostly disease-specific and related to dysfunction of the respective target tissues. For both T1D and T2D, there was a decrease in β-cell function pathways (e.g., “regulation of gene expression in β-cells”, “insulin secretion and processing”) and in “maturity-onset diabetes of the young”, which includes many transcription factors (TFs) involved in the maintenance of the β-cell phenotype and function (e.g., *PDX1* and *PAX6*) (Figures 1B, 1C, S1A, and B). Neuronal function pathways, including “neurotransmitter release cycle”, “neurexins and neuroligins”, and “transmission across chemical synapses”, were depleted in AD (Figure 1E). Inhibition of mitochondrial function pathways, including “mitochondrial protein import”, “respiratory electron transport”, “tricarboxylic acid cycle” and “oxidative phosphorylation” was common to AD and T2D (Figures 1C,1E, S1B, and D). Furthermore, lipid metabolism pathways (e.g., “peroxisome”, “steroid and cholesterol biosynthesis”) were downregulated in MS (Figures 1D and S1C), whereas “fatty acid elongation” and “propanoate and butanoate metabolism” were inhibited in T2D (Figure S1B).

Analysis of TF binding sites in promoter regions (transcription start site ± 2 kb) of differentially expressed genes for each disease identified a clear enrichment of IFN-induced TFs in the upregulated genes of T1D, including IFN regulatory factor 1 (IRF1), IRF2, and IRF8 (Figure S2A), which is in line with the marked induction of IFN-related pathways in T1D (Figure 1B). These TFs also appeared in islets from T2D patients (Figure S2B). REST-NRSF (neuron-restrictive silencer factor) was identified as the unique TF that binds to a set of downregulated genes in AD and as the top TF for T2D downregulated genes (Figures S2B and S2D).

We next investigated the overlap between significantly modified genes (either up- or downregulated) of the four diseases, using a false discovery rate <0.1 cutoff (Figure S3). There were 229 common genes between T1D and T2D, but less than 100 genes overlapped between the two types of diabetes and the other two diseases. Through a hypergeometric test using Reactome and KEGG databases as references, “cytokine signaling”, “interleukin signaling”, “interferon-γ signaling” and “type 1 diabetes” pathways were commonly upregulated in two or three diseases (Table S2A). Of note, there were only two commonly upregulated genes (i.e., *MS4A7* and *MSR1*) between the four diseases (Table S2A). The function of commonly downregulated genes between T1D and T2D were identified as neuronal function-related (e.g., “neurotransmitter receptors”, “postsynaptic signal transmission” and “GABA receptor activation”), β-cell function-related (e.g., “regulation of gene expression in β-cells”) and energy metabolism-related (e.g., “integration of energy metabolism”) (Table S2B). Because these genes were filtered by a fixed statistical threshold, this type of analysis largely depends on the number of samples analyzed. Despite the limitation of this approach, the enriched pathways (for either up- or downregulated genes) between two or three diseases generally agree with the above-mentioned GSEA results.

### Rank-rank hypergeometric overlap-based pairwise analysis demonstrates similarities between the four diseases mostly related to inflammation

**Figure 2:**
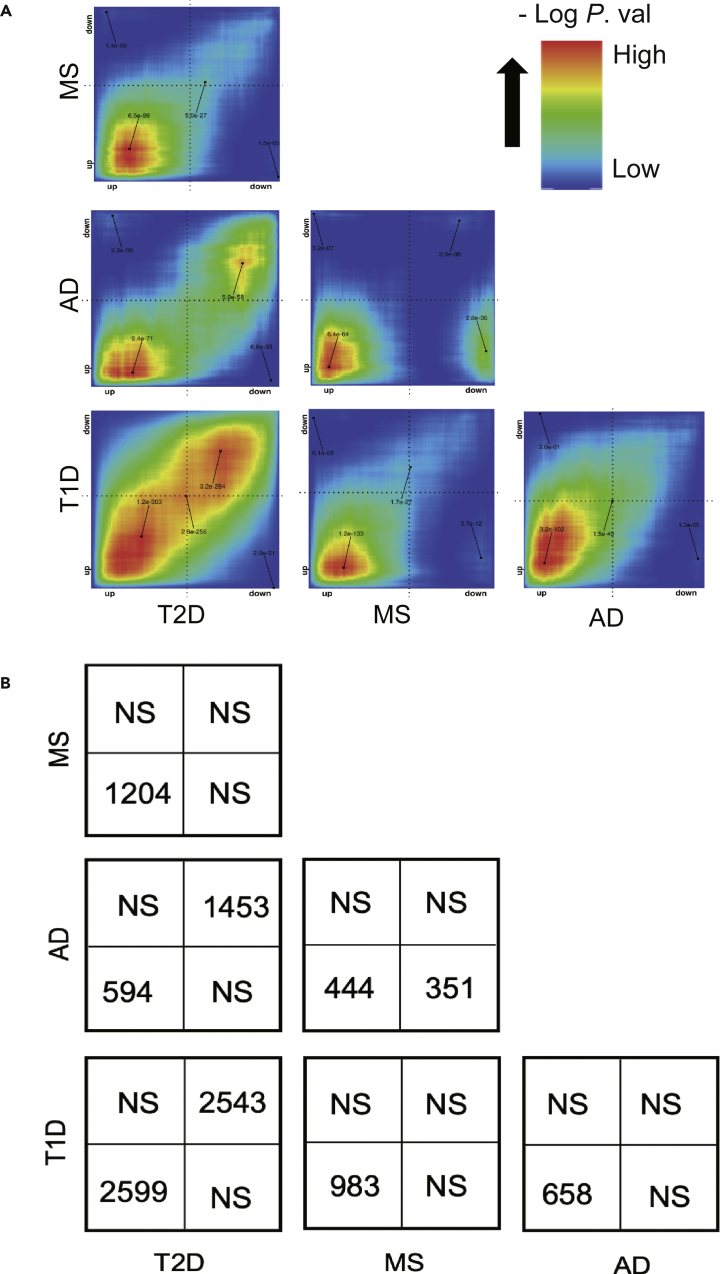
Pairwise rank-rank hypergeometric overlap (RRHO) analyses on the gene expression signatures of target tissues among the four diseases (A) The significance of the overlap between genes upregulated in both diseases (bottom left quadrant), downregulated in both (top right quadrant), upregulated in one and downregulated in another disease (top left or bottom right quadrants) is displayed by the level map with colors representing the -log adjusted p-values. (B) The panel displays the number of genes significantly overlapped in each pairwise analysis in (A). NS, not significant.

We next used the *rank-rank hypergeometric overlap* (RRHO) analysis^23^ to compare global transcriptomic signatures between the four diseases without the limitation of a fixed threshold (see STAR methods). We observed generally similar pairwise transcriptomic signatures between the four diseases, particularly for upregulated genes. The highest correlation was observed between T1D and T2D, both for up- and downregulated genes (Figure 2A). There was an unexpected and highly significant correlation between upregulated, but not downregulated, genes of T1D and AD, which is in line with the identification of “type 1 diabetes” as an upregulated pathway in AD (Figure S1D). In contrast, T2D presented a larger number of downregulated than upregulated genes in common with AD (Figures 2A and 2B).

**Figure 3:**
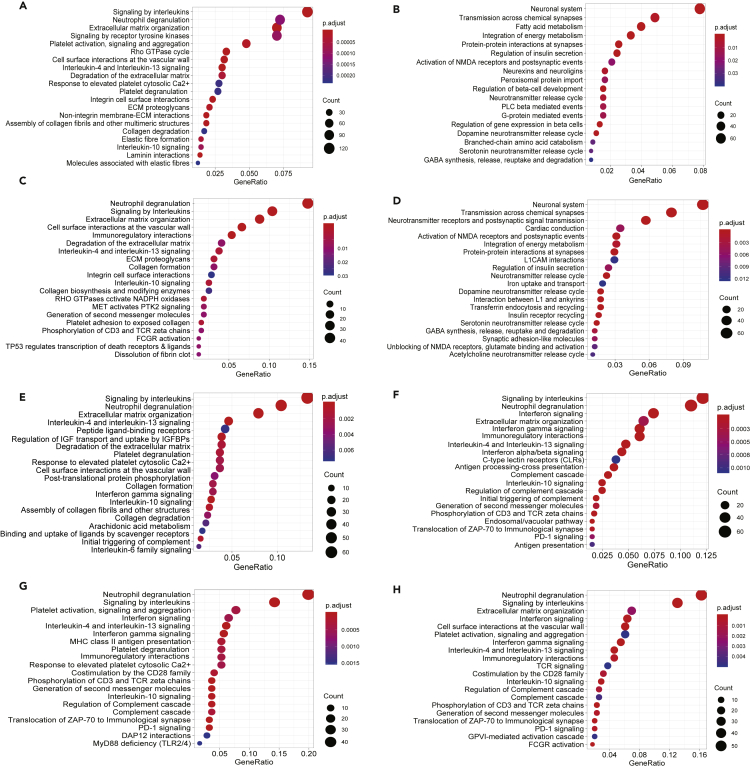
Functional enrichment analysis of overlapping genes among the four diseases (A–H) Genes significantly overlapping between different pairs of four diseases in the RRHO analysis were selected for functional enrichment analysis by Reactome database, using the R package *clusterProfiler*. The top 20 enriched pathways are displayed according to their adjusted p-values (Benjamini-Hochberg correction) and their gene ratio (number of modified genes/gene set size). Enriched pathways by genes significantly (A) upregulated both in T2D and T1D; (B) downregulated both in T2D and T1D; (C) upregulated both in T2D and AD; (D) downregulated in both T2D and AD; (E) upregulated in both T2D and MS; (F) upregulated in both MS and T1D; (G) upregulated in both MS and AD and (H) upregulated in both AD and T1D are shown (see also Figure S4).

Functional enrichment analysis of the genes in these overlapping RRHO-quadrants by Reactome database revealed that upregulation of “signaling by interleukins” and “extracellular matrix organization” and inhibition of “integration of energy metabolism”, “regulation of insulin secretion” and neuronal function-related pathways were common between “T1D and T2D” and “T2D and AD” (Figures 3A–3D). IFN signaling (and also “PD-1 signaling”, downstream of type I and type II IFNs^24^) was again identified as a commonly upregulated pathway between “T1D and AD”, “MS and AD”, and “MS and T1D” (Figures 3F–3H). The results by the KEGG database similarly identified enrichment of inflammatory components (e.g., “cytokine-cytokine receptor interaction”, “complement and coagulation cascades”, “TNF signaling”, “signaling by interleukins”, and “neutrophil degranulation”) in all pairwise combinations of the four diseases (Figures S4A, S4C, S4E, and S4H). Inhibition of “insulin secretion”, “fatty acid metabolism”, and “maturity-onset diabetes of the young” was again observed between “T2D and T1D” (Figure S4B), in keeping with disease-specific target tissue dysfunction. The downregulation of “cAMP signaling”, “insulin secretion”, and neuronal function-related pathways in “T2D and AD” (Figure S4D) highlights similarities of the two degenerative diseases.

We next investigated the potential upstream TFs orchestrating the inter-disease similarities by predicting the TF binding sites in the promoter region (transcription start site ± 2 kb) of commonly upregulated genes from the pairwise diseases comparison by RRHO (Figure 2). In line with the presence of IFN-related pathways (Figures 3E–3H), there was a clear enrichment of motifs for IFN-induced TFs, including IFN-stimulated response element (ISRE), IRF1, IRF3, IRF8, and type I IFN-stimulated response element (T1ISRE), when comparing MS to T1D or T2D and AD to MS or T1D (Figures S5E–S5H). Binding sites for NFκB-p65 were enriched for commonly upregulated genes between T1D and T2D, which is in line with the identification of NF-κB and TNF signaling in islet cells in these diseases (Figure S4A). For commonly downregulated genes from T2D vs T1D and T2D vs AD, we again identified REST-NRSF (Figures S5B and S5D), a transcriptional repressor of neural genes, which is in line with the downregulation of neuronal pathways (Figures 3B and 3D).

We next compared the differential expression of selected genes between the four diseases allocated visually by one of us (DLE) to potentially relevant functional groups. Many key genes belonging to antigen presentation were predominantly modified in the target tissues of T1D and to a lesser extent in AD and MS, but not in T2D (Table S3). Chemokines, cytokines, complement and IFNs were markedly upregulated in β-cells from T1D patients. Some genes related to granule release and synaptic cycle were downregulated in T2D and AD target tissues. There were few changes in autophagy, lysosomal degradation, free radical scavenging and DNA damage response genes. Several genes critical for β-cell function and belonging to glucose and lipid metabolism, protein translation and modification were affected in T2D and T1D. These observations were generally supported by functional enrichment performed individually or pairwise (Figures 1, S1, 3, and S4).

### Identification of potential therapeutic targets based on top concordant genes identified between diseases

**Figure 4:**
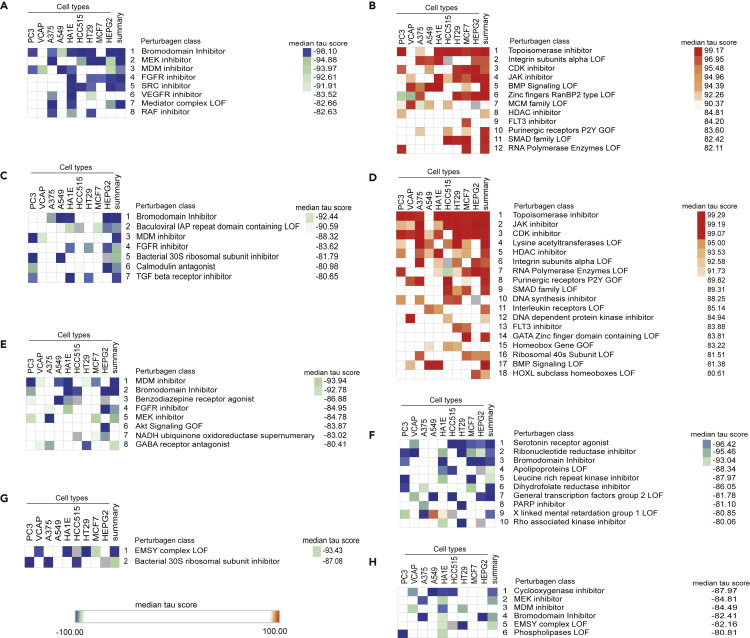
Identification of potential common therapeutic hits in the four diseases (A–H) The top 150 overlapping genes were submitted to the Connectivity Map database to identify perturbagen classes driving an opposite signature (negative and positive tau score for the up- and downregulated genes, respectively). Only classes with a median tau score <−80 (displayed in blue) and >80 (red) were considered. Predicted Perturbagen classes are displayed for (A) upregulated in T2D and T1D, (B) downregulated in T2D and T1D, (C) upregulated in T2D and AD, (D) downregulated in T2D and AD, (E) upregulated in T2D and MS, (F) upregulated in MS and T1D, (G) upregulated in MS and AD and (H) upregulated in AD and T1D.

To identify potential therapeutic targets, we compared the top 150 commonly up- or downregulated genes from the RRHO analysis against the cell signatures induced by chemical perturbations in the Connectivity Map (See STAR methods). We identified perturbagen classes driving opposite signatures to the ones we submitted, indicating that these chemical perturbations could reverse commonly altered pathways and have potential therapeutic use (Figures 4A–4H). The most consistently highly ranked perturbagen (often with |median *tau* scores| >90) was “Bromodomain inhibitor” when analyzing upregulated genes from the comparisons T2D versus T1D, T2D versus AD, T2D versus MS, MS versus T1D and T1D versus AD (Figures 4A, 4C, 4E–4F, and 4H). We have recently shown that two broad-action bromodomain inhibitors, namely I-BET151 and JQ1, prevent some of the deleterious effects of IFNα (a cytokine involved in the early steps of islet inflammation in T1D^10^^,^^25^) on human β-cells.^26^ SRC inhibitors and JAK inhibitors were further predicted as potential drugs for commonly perturbed genes in T2D and T1D (Figures 4A and 4B). Importantly, JAK inhibitors have been shown by us and others to protect human β-cells against pro-inflammatory cytokines^26^^,^^27^ and to prevent diabetes in mouse models.^27^ One of these JAK inhibitors, baricitinib, is presently being tested as a potential therapy for T1D (Clinical Trials.govNCT04774224).

**Figure 5:**
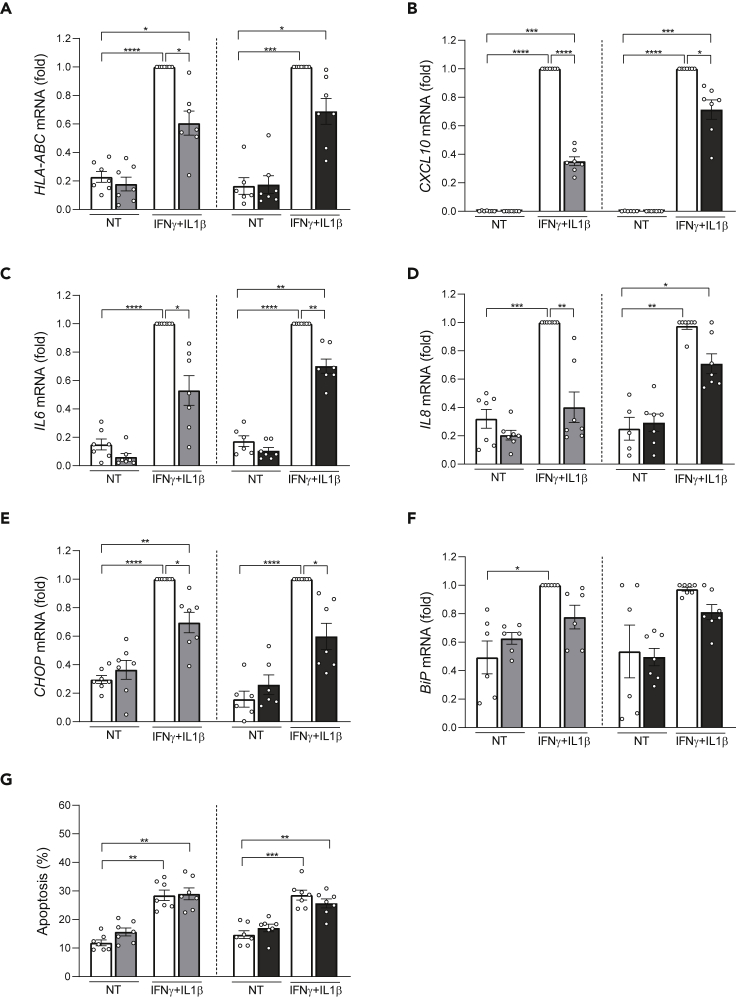
Bromodomain inhibitors attenuate cytokine-induced pro-inflammatory gene expression in human islets Human islets were pretreated for 6 h with the bromodomain inhibitors iBET-151 (1 μM, gray bars), GSK046 (1 μM, black bars) and then exposed to IFNγ (1000 U/mL) and IL1β (50 U/mL) or not (non-treated, NT, white bars) for 48 h. Ethanol (vehicle) and DMSO (vehicle) were used as respective controls for iBET-151 and GSK046. mRNA expression of *HLA-ABC(A)*, *CXCL10(B)*, *IL6(C)*, *IL8(D)* and the ER stress markers *CHOP(E)* and *BiP(F)* was analyzed by quantitative real-time PCR. Values were normalized to the geometric mean of the reference genes *β-actin* and *VAPA*, and the highest value of each experiment was considered as 1. (G) The percentage of apoptotic cells was counted after 48 h by Hoechst 33342 and propidium iodide staining. Results are mean ± SEM of 5–7 independent experiments. ∗p < 0.05, ∗∗p < 0.005, ∗∗∗p < 0.001 and ∗∗∗∗p < 0.0001 by ANOVA followed by Bonferroni correction for multiple comparisons (see also Figures S6 and S7).

**Figure 6:**
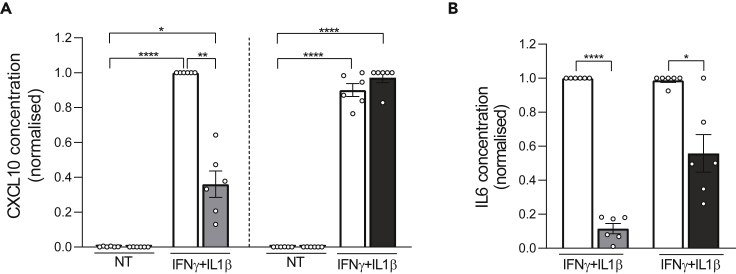
The bromodomain inhibitor iBET-151 reduces cytokine-induced chemokine and cytokine production (A and B) CXCL10 (A) and IL6 (B) protein release to the medium was quantified by ELISA. Human islets were pretreated for 6 h with the bromodomain inhibitors iBET-151 (1 μM, gray bars) or GSK046 (1 μM, black bars) and then exposed to IFNγ (1000 U/mL) and IL1β (50 U/mL) or not (non-treated, NT, white bars) for 48 h. Ethanol (vehicle) and DMSO (vehicle) were used as respective controls for iBET-151 and GSK046. The highest value of each experiment was considered as 1. IL6 was undetectable in non-treated (NT) conditions. Results are mean ± SEM of 6 independent experiments. ∗p < 0.05, ∗∗p < 0.005 and ∗∗∗∗p <0.0001 by paired Student’s *t*test for IL6 or ANOVA followed by Bonferroni correction for multiple comparisons for CXCL10.

Based on these findings, we evaluated the impact of a broad-action (I-BET151) and a more specifically inflammation-targeting (GSK046)^28^ bromodomain inhibitor in two models of human β-cell dysfunction in T1D and T2D, respectively the cytokines IFNγ + IL1β that contribute to β-cell apoptosis at more advanced stages of islet inflammation in T1D and the metabolic stressor palmitate that contributes to β-cell dysfunction and death in T2D.^10^^,^^16^^,^^29^^,^^30^^,^^31^ Exposure of human islets to IFNγ + IL1β for 48 h induced the mRNAs encoding for HLA-ABC, the chemokine CXCL10, the cytokines IL6 and IL8 and the endoplasmic reticulum (ER) stress markers CHOP and BiP (Figures 5A–5F). This was confirmed at the protein level, by measuring CXCL10 and IL6 accumulation in the medium (Figures 6A and B). IFNγ + IL1β also induced apoptosis (Figure 5G). The bromodomain inhibitors I-BET 151 and GSK046 reduced the pro-inflammatory effects of the cytokines and lowered CHOP expression (Figures 5A–5E, 6A, and 6B) but did not prevent apoptosis (Figure 5G). To investigate whether these effects take place at least in part at the β-cell level, we exposed the human β-cell line EndoC-βH1 to the same cytokines with or without the bromodomain inhibitors for 24 (Figures S6A–S6E) or 48 h (Figures S7A–S7E). The results were broadly similar to human islets, i.e., the bromodomain inhibitors partially prevented most cytokine-induced pro-inflammatory gene expression but did not prevent apoptosis.

**Figure 7:**
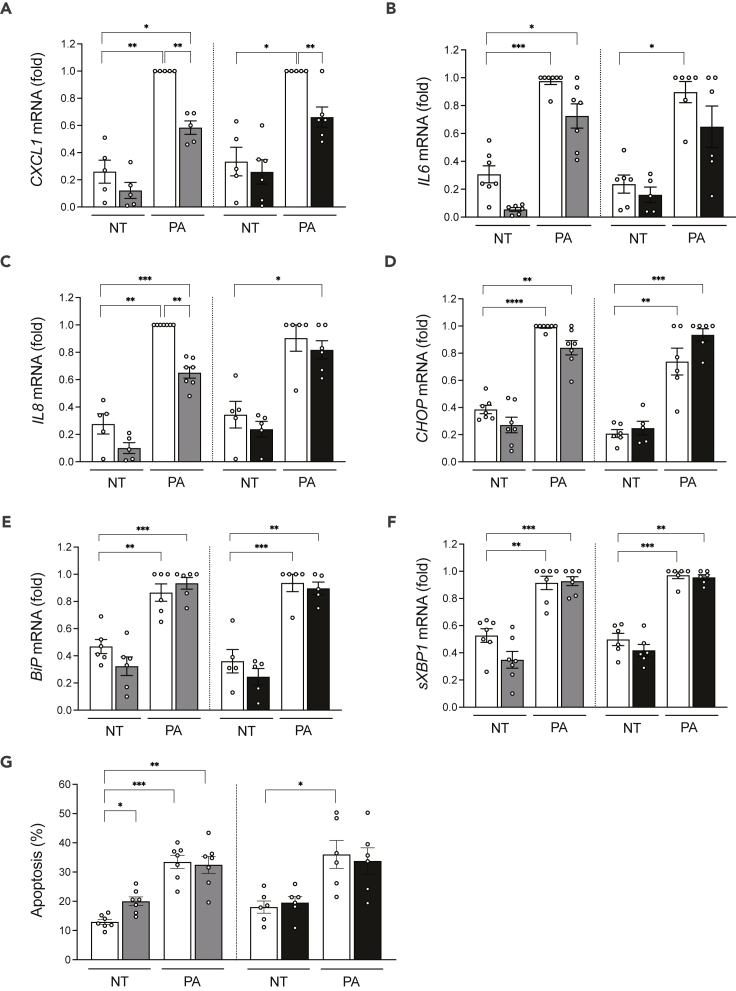
Bromodomain inhibitors attenuate some of the pro-inflammatory effects of palmitate in human islets Human islet cells were pretreated for 6 h with the bromodomain inhibitors iBET-151 (1 μM, gray bars) or GSK046 (1 μM, black bars) and then exposed to 0.5 mmol/L palmitate (PA) or not (non-treated, NT, white bars) for 48 h. Ethanol (vehicle) and DMSO (vehicle) were used as respective controls for iBET-151 and GSK046. mRNA expression of *CXCL1(A)*, *IL6(B)*, *IL8(C)* and the ER stress markers *CHOP(D)*, *BiP(E)* and spliced *XBP1(F)* was analyzed by quantitative real-time PCR. Values were normalized to the geometric mean of the reference genes *β-actin* and *VAPA*, and the highest value of each experiment was considered as 1. (G) The percentage of apoptotic cells was counted after 48 h by Hoechst 33342 and propidium iodide staining. Results are mean ± SEM of 6–7 independent experiments. ∗p < 0.05, ∗∗p < 0.005, ∗∗∗p < 0.001, and ∗∗∗∗p < 0.0001 by ANOVA followed by Bonferroni correction for multiple comparisons.

We next evaluated whether bromodomain inhibitors could protect human islets against the metabolic stressor palmitate (Figures 7A–7G). These experiments were not undertaken in EndoC-βH1 cells because these cells are resistant to palmitate due to their high stearoyl CoA desaturase expression.^32^ In human islets, palmitate induced the chemokine *CXCL1*, *IL6* and *IL8* and *CHOP*, *BiP* and spliced *XBP1*, and apoptosis (Figures 7A–7G). The beneficial effects of the bromodomain inhibitors were less marked in the context of palmitate than with cytokines (Figures 5A–5G). There was less palmitate induction of *CXCL1* and *IL8 (*Figures 7A and 7C), and for the latter, the protection was observed with iBET-151 but not GSK046. The bromodomain inhibitors did not protect the human islet cells from ER stress (Figures 7D–7F) or apoptosis (Figure 7G).

## Discussion

In the present study we investigated the hypothesis that key gene expression changes, potentially related to inflammation, are commonly present in the target tissues of autoimmune or degenerative diseases affecting pancreatic β-cells, namely T1D and T2D, and the brain, namely MS and AD. Exposure to different forms of stress leaves “molecular signatures” at the target tissue levels, and discovering similar gene signatures may allow the identification of key pathways to be targeted for therapy via drug repositioning or discovery.^14^^,^^25^

The transcriptome analysis of the target tissues in the four diseases showed concordant upregulation of cytokine-cytokine receptor interaction, chemokine signaling, and JAK-STAT signaling, supporting the idea that these diseases contain similar inflammatory molecular components (Figure S1). The IFNγ-regulated pathways, an indicator of adaptive immunity, were found augmented in T1D, MS, and AD, but not in T2D. This is in line with previous studies, showing that both innate and adaptive immunity are essential for developing MS^33^ and that neuroinflammation and innate immunity are hallmarks of AD.^34^ Adaptive immunity and its interactions with microglia are important for restraining AD through limiting amyloid pathology^35^ but at later phases of the disease may contribute to neuronal damage. Regarding T1D, the complex interaction between β-cells and innate/adaptive immune cells is critical for the development of the disease.^10^^,^^36^ Of note, the most intense expression of inflammatory markers in target tissue was observed in T1D, suggesting a more important impact of inflammation in this disease as compared to MS, T2D and AD. The present data could be confounded by immune cells infiltrating the target tissues, which could impact gene expression of inflammation/adaptive immunity pathways. Expression of the leukocyte marker CD45 was, however, low in the four tissues studied (Table S1), and IFN signatures are present in purified β-cells and neurons in T1D and MS, respectively^7^. Specific β-cell function pathways were downregulated in T1D and T2D, whereas neuronal function pathways were downregulated in AD. These changes imply dysfunction of the target tissues.

The functional enrichment for RRHO-quadrant genes between diseases cross-validated these molecular alterations, especially for the highly concordant upregulation of inflammatory pathways. As potential upstream regulators, we predicted many inflammation-induced TFs (e.g., IRFs and NF-κB-p65) for commonly upregulated genes in most combinations of the four diseases. Of interest, the TF REST-NRSF was predicted as an important regulator for commonly downregulated genes between T2D, AD, and T1D, which is in line with the striking depletion of neuronal function pathways in the pairwise comparisons between the three diseases. NRSF represses pancreatic endocrine and neuronal development and function through the recruitment of multiple transcriptional and epigenetic co-regulators that restrict endocrine or neuron fate acquisition; its expression wanes as these cell types differentiate.^37^^,^^38^^,^^39^

Based on the molecular changes shared between the four diseases, we mined *in silico* for drug repurposing strategies using the Connectivity Map L1000 platform,^14^ which includes pre-computed differential gene expression signatures from nine core cell lines exposed to chemical or genetic perturbations. The anti-correlation between highly concordant signatures of target tissues and Connectivity Map libraries allowed us to identify perturbagen classes that could target more than one disease (Figure 4). Among them, JAK inhibitors, acting downstream of types I and II IFN receptors, block the activation of JAK1 and JAK2 kinases. The JAK inhibitor baricitinib protects pancreatic β-cells against the deleterious effects of type I IFNs,^26^ and it is being tested for the prevention of T1D (Clinical Trials.govNCT04774224). *Src* family tyrosine kinase is expressed at high levels in cells specialized for exocytosis, such as neuronal and endocrine cells. It has been shown that PP2, an *Src* family tyrosine kinase inhibitor, enhances neurotransmitter release from neuronal cells.^40^ Two structurally different *Src* family kinase inhibitors, SU-6656 and PP2, enhanced Ca^2+^-dependent insulin secretion in rat pancreatic islets and INS-1 cells.^41^ The enriched “neurotransmitter release cycle” pathway for commonly downregulated genes from T2D and AD (Figure 3D) suggests that indeed the use of Src inhibitors could be beneficial for both diseases. The bromodomain and extra-terminal domain family of epigenetic reader proteins regulate inflammatory and cancer-related gene expression.^42^ Bromodomain inhibitors are being tested as a potential therapy in experimental models of AD,^43^ diabetes^44^ and MS.^45^ Treatment with JQ1 in 3-month-old mice carrying 3 mutations associated with familial Alzheimer’s disease reduced neuroinflammation, with decreased expression of pro-inflammatory modulators (e.g., IL-1β, Il-6 and TNFα).^43^ A short treatment with iBET-151 in non-obese diabetic mice prevented both insulitis and diabetes.^44^ Furthermore, JQ1 treatment in mice with experimental autoimmune encephalomyelitis (a model of MS) significantly protected them from encephalomyelitis by selectively preventing the generation of T_H_17 cells, essential effectors of autoimmunity in this model.^45^ Finally, the BET protein inhibitor Apabetalone decreased the *ex vivo* inflammatory responses of monocytes obtained from patients with type 2 diabetes/cardiovascular diseases.^46^ We have shown that iBET-151 prevents IFNα-induced inflammatory pathways but not apoptosis in human islets.^26^ GlaxoSmithKline has recently described the inhibitor GSK046 (targeting the second bromodomain) that is particularly effective in models of inflammatory and autoimmune diseases, including psoriasis, collagen-induced arthritis and non-alcoholic fatty liver disease.^28^ iBET-151 and GSK046 significantly protected human β-cells from the pro-inflammatory but not from the pro-apoptotic effects of IFNγ + IL1β (Figures 5, S6, and 7), cytokines that mimic advanced inflammatory features in T1D, present in the later stages of insulitis.^10^ There was a milder protective effect of the bromodomain inhibitors against palmitate-induced chemokine production, but they did not alter palmitate-induced ER stress or apoptosis (Figure 7). These agents may hence be more useful in settings of autoimmune β-cell destruction.

In conclusion, we integrated the transcriptomes of target tissues from four major diseases affecting β-cells or the brain. We identified commonly dysregulated gene signatures and mined these for potential therapeutic candidates. We validated i-BET151 and GSK046 as promising drugs to rescue pancreatic β-cells from aggressive inflammation in diabetes.

### Limitations of the study

A limitation of the present study is that the original RNA-seq datasets were generated in different studies, using different RNA-seq methods, and obtained from patients of different ages and sex. Due to differences in disease prevalence and the difficult access to target tissues, we only had 4–5 samples of target tissues of individuals affected by T1D or MS as compared to larger numbers for T2D (28 individuals) and AD (122). This may decrease the power of the analysis and lead to fewer modified genes being identified in T1D and MS. Despite these limitations, we identified disease-specific gene expression signatures - mostly related to downregulated genes - and commonly upregulated gene signatures - mostly related to inflammation - in the four diseases.

We acknowledge that the study only tested i-BET151 and GSK046 on human beta-cells *in vitro* and that a follow-up *in vivo* validation study is needed. It also remains to be tested whether similar beneficial effects can be observed for neuronal protection in MS and AD.

## STAR★Methods

### Key resources table

**Table:** 

REAGENT or RESOURCE	SOURCE	IDENTIFIER
**Chemicals, peptides, and recombinant proteins**		

I-BET-151 (1 μM)	Selleckchem, Munich, Germany	Cat# S2780
GSK046 (1 μM)	MedChemExpress, Monmouth Junction, USA	Cat# HY-136571
IFN-γ (1000 U/mL)	PeproTech, Rocky Hill, NJ, USA	Cat# 300–02
IL1β (50 U/mL)	R&D Systems, Minneapolis, MN, USA	Cat# 201-LB-005
Palmitate	Sigma-Aldrich, Saint Louis, USA	Cat# P5585-10G
BSA	Roche, Neuilly-sur-Seine, Basal, Switzerland	Cat# 10775835001
Ham’s F-10 medium	Thermo Fisher Scientific, grand island, NY, USA	Cat# 41550–021
Propidium iodide (10 μg/mL)	Sigma Aldrich, Saint Louis, USA	Cat# P4170-100MG
Hoechst 33342 (10 μg/mL)	Sigma Aldrich, Saint Louis, USA	Cat# 14533–100MG

**Critical commercial assays**		

Dynabeads mRNA DIRECT purification kit	Invitrogen, Carlsbad, CA, USA	Cat# 61012
Reverse Transcriptase Core kit	Eurogentec, Liège, Belgium	Cat# RT-RTCK-05
CXCL10 ELISA Kit	Quantikine ELISA kit, R&D Systems, Minneapolis, MN, USA	Cat# DIP100
IL6 ELISA kit	Quantikine ELISA kit, R&D Systems, Minneapolis, MN, USA	Cat# D6050

**Deposited data**		

Gencode release 36 (GRCh38.p13) gft	The GENCODE project	https://www.gencodegenes.org
Gencode release 36 (GRCh38.p13) fasta	The GENCODE project	https://www.gencodegenes.org
Genome Reference Consortium Human Build 38 (GRCh38)	Genome Reference Consortium	https://www.ncbi.nlm.nih.gov/grc/human
Molecular Signatures Database	The joint project of UC San Diego and Broad Institute	https://www.gsea-msigdb.org/gsea/msigdb/
Human islet purified β-cells RNA-seq	Gene Expression Omnibus	GSE121863
Human islet RNA-seq	Gene Expression Omnibus	GSE50244
Human islet RNA-seq	Gene Expression Omnibus	GSE159984
Human islet RNA-seq	EUROPEAN GENOME-PHENOME ARCHIVE	EGAS00001005535
Human brain RNA-seq	Gene Expression Omnibus	GSE100297
Human brain RNA-seq	SYNAPSE platform	Syn21589959

**Experimental models: Cell lines**		

Human EndoC-βH1	Dr. R. Scharfmann	Inserm U1016, CNRS UMR8104, F-75014

**Experimental models: Organisms/strains**		

Human islets	Pisa University, Italy	N/A

**Oligonucleotides**		

Primers for Figures 5, 7, S6, and S7, see Table S5	Eurogentec, Liège, Belgium	N/A

**Software and algorithms**		

Salmon V1.4.0	Patro et al., 2017^47^	https://combine-lab.github.io/salmon/
deg-rrho-gsea	This paper	https://doi.org/10.5281/zenodo.7018833
R package DESeq2 V1.28.1	Love et al., 2014^48^	http://bioconductor.org
Rank-Rank Hypergeometric Overlap	University of California, Los Angeles, United States	https://systems.crump.ucla.edu/rankrank/
R package clusterProfiler V3.12.0	YuLab-SMU, Guangzhou, China	https://github.com/YuLab-SMU/clusterProfiler
fGSEA V1.20.0	Korotkevich et al., 2021^49^	https://www.biorxiv.org/content/10.1101/060012v3.full
HOMER V4.11	University of California, San Diego, United States	http://homer.ucsd.edu/homer/
Connectivity Map L1000 platform	Broad institute, Cambridge, United States	https://clue.io/query

**Other**		

GraphPad Prism 9 software	GraphPad Software, La Jolla, CA, USA	N/A

### Resource availability

#### Lead contact

Further information and requests for resources and reagents should be directed to and will be fulfilled by the Lead Contact, Decio L. Eizirik (decio.laks.eizirik@ulb.be).

#### Materials availability

This study did not generate new unique reagents.

### Experimental model and subject details

#### Human pancreatic islets

Human islets from 8 non-diabetic organ donors (Table S4) were isolated before November 2021 by enzymatic digestion and density-gradient purification,^50^ with the consent of the local Ethical Committee in Pisa, Italy. Islets were cultured in M199 medium (5.5 mmol/L of glucose) and sent to Brussels, Belgium, where they were dispersed.^51^ The percentage β-cells in the human islet preparations was 52 ± 16%, determined by insulin immunofluorescence.^52^^,^^54^

#### Cell lines

The human β-cell line EndoC-βH1 was provided by Dr. R. Scharfmann^53^ (Université deParis, Institut Cochin, Inserm U1016, CNRS UMR8104, F-75014, Paris, France) and cultured in Matrigel fibronectin-coated plates as previously reported.^54^

### Method details

#### Culture and treatment of human EndoC-βH1 cells and human islet cells

EndoC-βH1 cells and dispersed human islet cells were pretreated for 6 h with two chemical inhibitors of bromodomain and extra-terminal family proteins, namely I-BET-151 (1 μM; Selleckchem, Munich, Germany) and GSK046 (1 μM; MedChemExpress, Monmouth Junction, USA), or their respective vehicles, i.e., ethanol and DMSO. EndoC-βH1 cells and dispersed human islet cells were exposed to a combination of two human pro-inflammatory cytokines, IFN-γ (1000 U/mL; PeproTech, Rocky Hill, NJ, USA) and IL1β (50 U/mL; R&D Systems, Minneapolis, MN, USA) for 24 or 48 h. Dispersed human islet cells were exposed to palmitate (Sigma-Aldrich, Saint Louis, USA) for 48 h. Palmitate was administered to the cells as a conjugate with 7.5% fatty acid-free BSA (Roche, Neuilly-sur-Seine, Basal, Switzerland) to obtain a palmitate stock solution of 5 mmol/L (ratio 1:4.5)^51^. The palmitate stock solution was diluted in Ham’s F-10 medium in presence of 5.6 mmol/L glucose (Thermo Fisher Scientific, Grand Island, NY, USA) to obtain a 0.5 mmol/L final concentration at a fixed concentration of 0.75% BSA. Unconjugated BSA was used as the non-treated control. These concentrations were selected based on our previous studies.^55^^,^^56^^,^^57^

#### mRNA extraction and quantitative real-time PCR

Polyadenylated mRNA was isolated from cultured cells using the Dynabeads mRNA DIRECT purification kit (Invitrogen, Carlsbad, CA, USA) following the manufacturer’s instructions. mRNA was reverse transcribed using the Reverse Transcriptase Core kit (Eurogentec, Liège, Belgium). Quantitative real-time PCR was performed using SYBR Green and data were expressed as number of copies/μL using a standard curve. Gene expression was corrected by the geometric mean of the reference genes *β-actin* and *VAPA*, as their expression is not modified under the experimental conditions used here.^58^ The highest value of each experiment was considered as 1. Primers sequences are listed in Table S5.

#### ELISA

Supernatants of dispersed human islet cells (30,000 cells/200 μL) pretreated with the bromodomain inhibitors IBET-151 or GSK046 and exposed or not to IFN-γ + IL1β were used to determine CXCL10 and IL6 secretion to the medium by ELISA (Quantikine ELISA kit, R&D Systems, Minneapolis, MN, USA).

#### Assessment of apoptosis

The percentage of viable, apoptotic, and necrotic cells was assessed by microscopy after nuclear dye staining (propidium iodide, 10 μg/mL, and Hoechst 33342, 10 μg/mL, Sigma-Aldrich, St. Louis, MO, USA). A minimum of 500 cells was counted for each experimental condition by two different observers, one of them unaware of sample identity.

### Quantification and statistical analysis

#### Quality control, quantification, and differential analysis of RNA-seq data

Raw RNA-seq data of target tissues from T1D,^15^ T2D,^16^^,^^17^^,^^18^ MS^19^ and AD^59^ were gathered from the Gene Expression Omnibus (GEO) Portal, European Genome-Phenome Archive (EGA) and Synapse Platform (Table 1). For each dataset, the raw RNA sequencing reads in Fastq format were processed with *fastp 0*.*19*.*6*^60^ using the default parameters for quality control, adaptor trimming, and quality filtering to obtain clean reads for downstream analysis. Gene expression levels of target tissues were quantified as TPM with *Salmon 1*.*4*.*0*^47^ using additional parameters “--seqBias--gcBias--validateMappings” to remove potential sequencing bias. The transcriptome reference was based on the indexed GENCODE version 36 (GRCh38.p13)^61^ with the default *k*-mer values. Differential analysis was performed by *DESeq2 1*.*28*.*1.*^48^ There was a relatively even distribution of age and sex between cases and controls. Because the T2D cohort was collected from three independent studies^16^^,^^17^^,^^18^ and AD cohorts were sequenced from three separate batches of samples,^59^ we applied batch correction in the general linear model used in DESeq2, taking batch as a confounding factor and formulated the design matrix (design = ∼ batch + condition) to estimate the dispersions and the log_2_ fold changes of the model. All other parameters used in the differential analysis of the diseases were similar. After correction of batch effects, a log_2_ fold change was computed and a *Wald test* was assessed with a p-value and an adjusted p-value (Benjamini-Hochberg correction) for differential analysis by DESeq2. The threshold to determine a gene as differentially expressed was adjusted p-value <0.05.

#### Functional enrichment using GSEA or hypergeometric test

GSEA was based on pre-ranked Wald statistics (the ratio of log_2_ fold change and the SE of estimation) generated from the DESeq2 pipeline. The *fGSEA* algorithm^49^ was performed against the Reactome^21^ and KEGG^22^ databases. The number of permutations was set as 50,000 for the most accurate p-values and the gene sets, including the number of genes between 15 and 500, were chosen as references. Significantly enriched pathways (adjusted p-value <0.05, Benjamini-Hochberg correction) were then sorted according to their normalized enrichment score (NES). To decide the functional enrichment of genes significantly overlapped in RRHO pairwise analysis, we conducted a hypergeometric test incorporated in *clusterProfiler (3*.*12*.*0)* tool^62^ against the Reactome and KEGG databases for genes with common up- or downregulation. Pathways with adjusted p-values <0.05 (Benjamini-Hochberg correction) were considered significantly enriched.

#### Rank-rank hypergeometric overlap (RRHO) pairwise analysis

To compare the global transcriptomic signatures of target tissues, we applied the RRHO algorithm,^23^ an unbiased and threshold-free method to reveal similarities and dissimilarities between diseases. For each pair of diseases, genes measured in both experiments were ranked according to their log_2_-transformed fold-change generated by DESeq2, from the most up-to the most downregulated ones. A hypergeometric test was performed to assess the significance of the similarity of gene profile, using a sliding window with step size (i.e., 50) for each pair of diseases. A False Discovery Rate correction was applied to adjust for the multiple hypothesis testing. The visualization of the output of this analysis is the RRHO level map (Figure 2A), in which the most significant hypergeometric p-value (log_10_ transformed and direction-signed) was labeled after computing all possible rank combinations, generating an index of the matrix for the most significant rank combination in each pair of diseases. Based on the hypergeometric test, we defined the most significant commonly regulated genes as the intersected genes above the most significant rank combination (with the most significant hypergeometric p-value). The RRHO level map is visualized as a heatmap displaying the degree of the similarities or dissimilarities in quadrants (e.g., commonly up- or downregulated in two diseases, upregulated in one disease and downregulated in the other).

#### TF binding site discovery

TF binding sites were searched with *HOMER* software^63^ in the promoter regions of genes from up- or downregulated genes for each disease or the commonly up- or downregulated genes in a disease pair identified by RRHO. The promoter regions were defined as the ± 2,000 base pairs from the transcription start sites of these genes. We used the script *findMotifs*.*pl* incorporated in *HOMER* with the parameters “--start--2000--end 2000--length 8,10,12”. TFs predicted with an enrichment p-value <0.05 by a hypergeometric test were considered significant.

#### Identification of potential therapeutic targets

To identify potential therapeutic targets for pairs of diseases, we selected the top 150 most up- or downregulated genes from the RRHO common gene set and submitted them to the Connectivity Map L1000 platform^14^ through the cloud-based CLUE platform (https://clue.io). The gene signatures revealed from our datasets were matched with the ones included in Connectivity Map libraries, which contain gene signatures for cells under many chemical or genetic perturbations. This allowed us to search for potential drugs that could restore the differential transcriptomes for more than one disease.

#### Statistical analysis for the human β-cell experiments

Data are expressed as means ± SEM EndoC-βH1 cells from different passages or human islets from different donors were considered as independent experiments. Differences between experimental conditions were assessed by Student’s paired t-test or one-way ANOVA or linear mixed model in case of missing values, followed by Bonferroni correction for multiple comparisons as indicated in the figure legends. Results with p-value ≤0.05 were considered significant. Analyses were performed using GraphPad Prism 9 software (GraphPad Software, La Jolla, CA, USA).

## Data Availability

• RNA sequencing data used in this article are all publicly available. Accession numbers are listed in the key resources table. • The code for analyses has been deposited at Zenodo and is now publicly available. DOI is listed in the key resources table. • Any additional information required to re-analyze the data reported in this article is available from the lead contact on request.

## Supplemental information

Document S1. Figures S1–S7, Tables S1, S4, and S5

Table S2. Commonly regulated genes between different combinations of the four diseases, related to Figures 1 and S3

Table S3. Classification of selected disease-modulated genes into selected functional groups, related to Figure 1
