# Supplementary material for: Mining the transcriptome of target tissues of autoimmune and degenerative pancreatic β-cell and brain diseases to discover therapies
Source: iScience. 2022 Oct 17;25(11):105376. doi: 10.1016/j.isci.2022.105376 (PMC9636054; doi:10.1016/j.isci.2022.105376)
Supplement: Document S1. Figures S1–S7, Tables S1, S4, and S5 [file mmc1.pdf]

iScience, Volume *25*

Supplemental information

Mining the transcriptome of target tissues of autoimmune and degenerative pancreatic β-cell and brain diseases to discover therapies

Xiaoyan Yi, Bianca Marmontel de Souza, Toshiaki Sawatani, Florian Szymczak, Lorella Marselli, Piero Marchetti, Miriam Cnop, and Decio L. Eizirik

Supplementary Information

Supplementary Figures

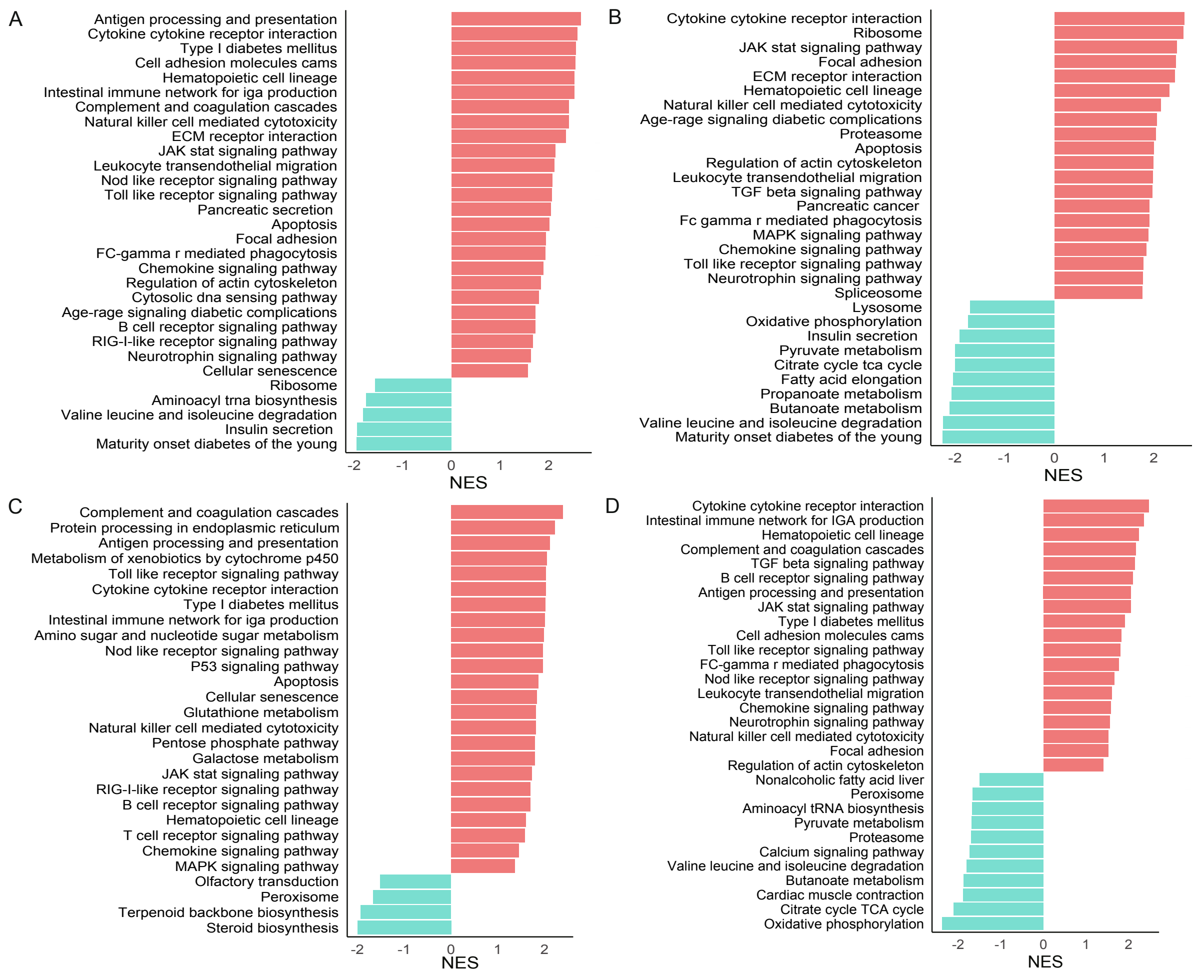

Figure S1. Overview of the enriched top 30 KEGG signaling pathways in the target tissues of the four diseases, related to Figure 1. Gene set enrichment analysis (GSEA) of (A) T1D, (B) T2D, (C) MS, and (D) AD using the KEGG database. Bars in red and blue represent positive and negative enrichment in the associated pathways, respectively. The x-axis shows the normalized enrichment score (NES) of the *fGSEA* analysis, and the y-axis shows the enriched pathways with an adjusted *P*-value <0.05.

A

T1D upregulated genes

Motif	Name	P-value	% of Target Sequences with Motif	% of Background Sequences with Motif
CAGTTTCACTTTGACTCA	bZIP:IRF	1.00E-04	46.13%	35.42%
GAAAGTGAAAGT	IRF2	1.00E-04	19.64%	11.97%
GGAAGTGAAAGT	IRF8	1.00E-03	44.35%	35.09%
GAAAGTGAAAGT	IRF1	1.00E-03	23.21%	15.96%
GGAAGTGAAAGT	PU.1:IRF8	1.00E-03	30.36%	22.57%
ACTTTCGTTTCT	T1ISRE	1.00E-02	3.57%	1.22%
CGCTGGCCCCCAGATAA	GATA:SCL	1.00E-02	17.86%	12.08%
ATTTTCCATT	NFAT	1.00E-02	74.40%	66.73%
AGCAGCTGCTGC	MyoD	1.00E-02	74.40%	67.06%
AAAGAGGAAGTG	SpiB	1.00E-02	35.42%	28.15%

T1D downregulated genes

Motif	Name	P-value	% of Target Sequences with Motif	% of Background Sequences with Motif
AACCTTCGATGGCTCCCTATTGCCG	ZNF16	1.00E-03	6.15%	1.54%
ATGATGCAAT	Atf4	1.00E-02	34.62%	22.59%
ATTTTCCATT	NFAT	1.00E-02	78.46%	66.29%
CCATTGTATGCAAAT	Oct4:Sox17	1.00E-02	21.54%	12.36%
GGAGCTGTCCATGGTGCTGA	REST-NRSF	1.00E-02	5.38%	1.41%
ATCAATCAAT	Cux2	1.00E-02	46.92%	34.69%
GCTGTGGTTT	RUNX-AML	1.00E-02	65.38%	54.57%

B

T2D upregulated genes

Motif	Name	P-value	% of Target Sequences with Motif	% of Background Sequences with Motif
GAAAGTGAAAGT	IRF2	1.00E-05	18.14%	11.81%
GAAAGTGAAAGT	IRF1	1.00E-05	22.71%	15.72%
CCAGCTGTTG	Twist2	1.00E-05	94.79%	89.76%
ATTTGCATAACAATG	OCT4-SOX2-TCF -NANOG	1.00E-05	22.56%	15.90%
ACCATCTGTT	NeuroG2	1.00E-04	91.96%	86.44%
AGCAGCTGCTGC	MyoD	1.00E-04	75.08%	67.48%
GACCAGCTGT	BHLHA15	1.00E-04	89.75%	84.10%
TAACAGCTGG	Tcf21	1.00E-04	80.60%	73.89%
GGAAGTGAAAGT	IRF8	1.00E-04	42.11%	34.65%
GCCATCTGGC	TCF4	1.00E-04	91.01%	85.91%

T2D downregulated genes

Motif	Name	P-value	% of Targets Sequences with Motif	% of Background Sequences with Motif
GGAGCTGTCCATGGTGCTGA	REST-NRSF	1.00E-05	4.22%	1.23%
AGTGGGCGGAGC	Sp5	1.00E-03	89.36%	83.91%
GGGGCCCCGCCCC	Sp2	1.00E-03	94.58%	90.41%
ATCAATCAAT	Cux2	1.00E-03	46.39%	39.03%
GGTGGGCGTGGC	KLF14	1.00E-03	95.78%	91.98%
TGGGGAAGGGCA	ZNF467	1.00E-03	82.13%	76.00%
GGTGCTGACAGC	Tbx20	1.00E-02	26.31%	20.49%
GGCAGTTA	MYB	1.00E-02	95.38%	92.04%
ATATTGATCC	HNF6	1.00E-02	52.81%	46.41%
CCTTTGTT	Sox3	1.00E-02	89.56%	85.32%

C

MS upregulated genes

Motif	Motif Name	P-value	% of Target Sequences with Motif	% of Background Sequences with Motif
GTTTCCAGGAAG	STAT4	1.00E-03	81.06%	70.57%
TTCTAGAAGCTTCTA	HRE	1.00E-03	31.72%	21.63%
GATGACTCATCC	Atf3	1.00E-03	56.39%	45.20%
AAGGTGTGAA	Tbr1	1.00E-03	88.99%	80.57%
TTAAGTGGTT	Bapx1	1.00E-02	97.80%	93.01%
TATGACTCAT	BATF	1.00E-02	54.63%	44.32%
CAAGGTCACG	SF1	1.00E-02	50.22%	40.31%
GGATGACTCATC	Fra1	1.00E-02	49.78%	40.05%
GGATGACTCATC	Fos	1.00E-02	51.10%	41.55%
GATGACTCAT	JunB	1.00E-02	48.90%	39.56%

MS downregulated genes

Motif	Name	P-value	% of Target Sequences with Motif	% of Background Sequences with Motif
GGTCAGCTGT	Ap4	1.00E-06	87.16%	78.78%
TCTTATCTCT	Gata6	1.00E-05	71.28%	62.43%
ACAGCTGCTG	Tcf12	1.00E-04	80.24%	73.03%
AGATAAGA	GATA3	1.00E-04	85.64%	79.49%
AGATAAGATC	TRPS1	1.00E-04	92.23%	87.28%
GGGAACAATGG	Sox7	1.00E-03	33.61%	26.58%
ATGAATATTC	Pit1	1.00E-03	68.41%	61.05%
AACAGCTGG	Tcf21	1.00E-03	78.55%	71.96%
TATGCAAATGA	Brn1	1.00E-03	32.94%	26.23%
AGCAGCTGC	E2A	1.00E-03	91.05%	86.27%

D

AD upregulated genes

Motif	Name	P-value	% of Target Sequences with Motif	% of Background Sequences with Motif
CAGCCTGTGTAAACAGG	Fox:Ebox	1.00E-03	80.26%	68.11%
ATTTTCCATT	NFAT	1.00E-02	82.89%	72.51%
CACTTCCGGT	Fli1	1.00E-02	90.79%	82.29%
GAGCCGAGC	ZNF519	1.00E-02	38.16%	27.93%
GCCAATAAAA	Hoxd13	1.00E-02	93.42%	86.42%
AAAGTAAACA	FOXA1	1.00E-02	86.18%	78.03%
TTTTATTGGG	HOXB13	1.00E-02	84.21%	75.85%
CCGAAACAATGG	Sox7	1.00E-02	37.50%	28.25%
TGTTTATTTAGC	FoxD3	1.00E-02	76.32%	67.09%
AAGGTGGCGCAGGCA	ZNF165	1.00E-02	26.97%	18.85%

AD downregulated genes

Motif	Name	P-value	% of Target Sequences with Motif	% of Background Sequences with Motif
GGAGCTGTCCATGGTGCTGA	REST-NRSF	1.00E-02	4.97%	1.59%

Figure S2. Transcription factor binding site motif analysis reveals potential upstream regulators of the modified genes in the four diseases, related to Figure 1. The promoter regions (transcription start site ± 2kb) of the differentially expressed genes in each disease were analyzed using *HOMER* tools to predict known transcription factor binding site motifs. Tables show the sequence motifs, name, *P*-value and frequency (%) in target as well in background sequences. Top 10 transcription factor binding sites enriched in up- and downregulated genes of (A) T1D, (B) T2D, (C) MS and (D) AD are displayed

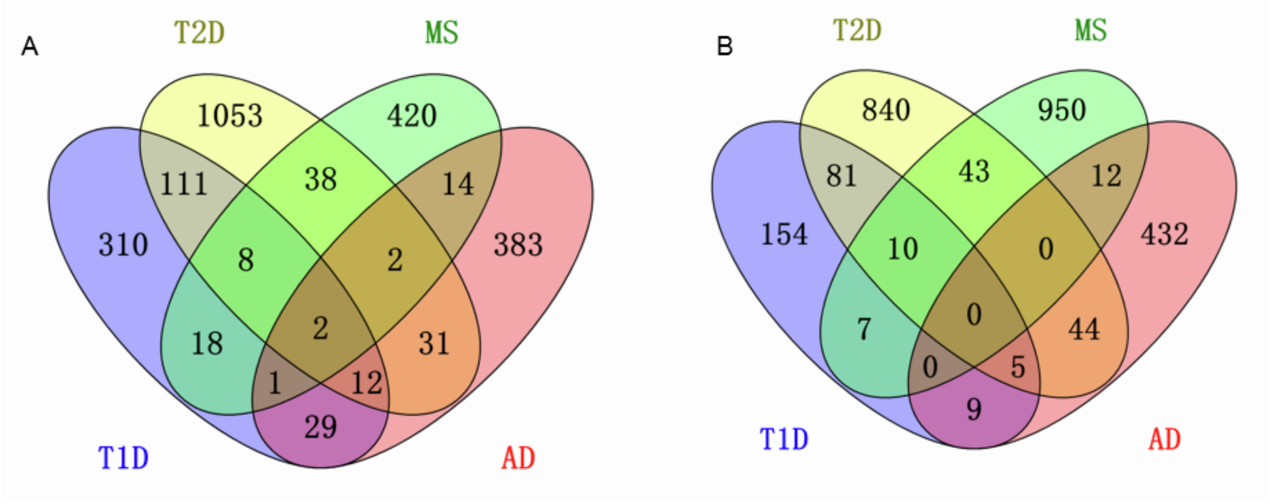

Figure S3: Venn diagrams of differentially expressed genes in T1D, T2D, MS and AD in bulk RNA-seq data, related to Figure 1. Venn diagrams of (A) upregulated and (B) downregulated genes in the four diseases. Differential expression was assessed with *DESeq2 1.28.1* and genes were selected with an adjusted *P*-value <0.10 (Benjamini Hochberg correction). Sample sizes for the data are as follows: T1D (n = 4 for patients, n = 10 for controls); T2D (n = 28 for patients, n = 183 for controls); MS (n = 5 for patients, n = 5 for controls; AD (n = 122 for patients, n = 80 for controls).

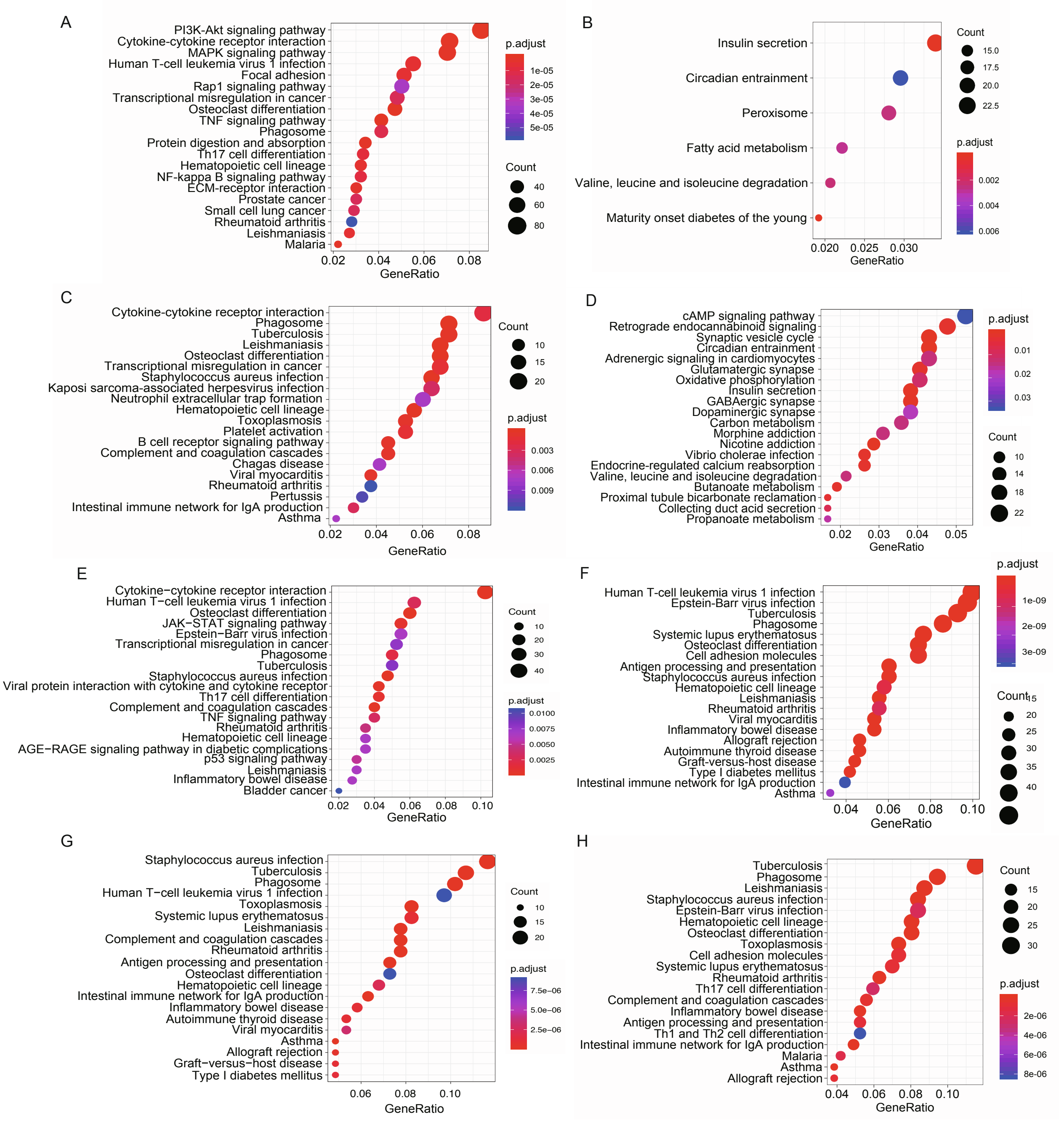

Figure S4. Functional enrichment analysis of overlapped genes among the four diseases, related to Figures 2 and 3. Genes significantly overlapped between different pairs of diseases detected in the RRHO analysis (Figure 2) were selected for enrichment analysis using the *clusterProfiler* via the KEGG database. The top 20 gene sets are represented according to their adjusted P-values (Benjamini Hochberg correction) and their gene ratio (no. of modified genes/gene set size). Enriched pathways by genes significantly (A) upregulated both in T2D and T1D, (B) downregulated both in T2D and T1D, (C) upregulated both in T2D and AD, (D) downregulated both in T2D and AD, (E) upregulated both in T2D and MS, (F) upregulated both in MS and T1D, (G) upregulated both in MS and AD, and (H) upregulated both in AD and T1D are displayed.

A

Motif	Name	P-value	% of Target Sequences with Motif	% of Background Sequences with Motif
	MafK	1.00E-04	30.00%	19.71%
	Tbx6	1.00E-02	85.00%	77.13%
	Fosl2	1.00E-02	35.77%	26.98%
	OCT4-SOX2-TCF-NANOG	1.00E-02	21.54%	14.62%
	Twist2	1.00E-02	95.38%	90.34%
	MafA	1.00E-02	68.46%	60.23%
	NF-E2	1.00E-02	8.08%	4.28%
	HIF2a	1.00E-02	48.08%	40.11%
	NFkB-p65-Rel	1.00E-02	12.69%	7.96%
	NFkB-p65	1.00E-02	65.77%	58.06%

B

Motif	Name	P-value	% of Target Sequences with Motif	% of Background Sequences with Motif
	ZNF416	1.00E-04	93.33%	83.44%
	Atoh1	1.00E-03	84.62%	74.07%
	REST-NRSF	1.00E-03	5.13%	1.33%
	Znf263	1.00E-03	98.46%	93.00%
	PGR	1.00E-02	29.74%	20.69%
	Olig2	1.00E-02	96.41%	90.68%
	Nr5a2	1.00E-02	66.67%	56.39%
	ZNF675	1.00E-02	24.10%	16.14%
	Pax7	1.00E-02	16.92%	10.24%
	Tbet	1.00E-02	81.03%	72.45%

C

Motif	Name	P-value	% of Target Sequences with Motif	% of Background Sequences with Motif
	Tlx	1.00E-03	50.38%	39.27%
	CEBP	1.00E-03	63.26%	52.85%
	Tcf12	1.00E-03	79.92%	70.88%
	SpiB	1.00E-03	37.88%	28.65%
	MITF	1.00E-02	71.21%	62.76%
	HRE	1.00E-02	24.24%	17.23%
	E2F	1.00E-02	14.02%	8.65%
	EWS:ERG-fusion	1.00E-02	71.97%	63.64%
	MyoD	1.00E-02	71.59%	63.63%
	HRE	1.00E-02	30.30%	23.16%

D

Motif	Name	P-value	% of Target Sequences with Motif	% of Background Sequences with Motif
	REST-NRSF	1.00E-10	8.79%	1.40%
	Olig2	1.00E-03	96.65%	90.84%
	ZNF652	1.00E-03	36.40%	26.32%
	ZNF519	1.00E-03	46.03%	35.47%
	Pit1	1.00E-02	37.24%	28.18%
	NFkB-p65	1.00E-02	65.69%	56.29%
	DUX4	1.00E-02	8.79%	4.36%
	Gfi1b	1.00E-02	59.83%	50.40%
	EAR2	1.00E-02	89.12%	82.39%
	Zfp281	1.00E-02	47.28%	38.83%

E

Motif	Name	P-value	% of Target Sequences with Motif	% of Background Sequences with Motif
	HRE	1.00E-03	31.42%	22.17%
	ISRE	1.00E-02	12.64%	7.32%
	Zfp809	1.00E-02	48.28%	39.18%
	STAT4	1.00E-02	79.31%	71.20%
	HRE	1.00E-02	24.90%	17.93%
	IRF1	1.00E-02	22.22%	15.69%
	VDR	1.00E-02	33.33%	26.20%
	MafA	1.00E-02	67.82%	60.15%
	Six1	1.00E-02	29.89%	23.19%

F

Motif	Name	P-value	% of Target Sequences with Motif	% of Background Sequences with Motif
	IRF8	1.00E-03	47.35%	35.58%
	SpiB	1.00E-03	38.78%	28.39%
	IRF3	1.00E-03	44.49%	33.83%
	NFkB-p65	1.00E-03	66.53%	56.35%
	Hoxd10	1.00E-02	80.41%	71.62%
	Bcl11a	1.00E-02	69.39%	60.54%
	IRF1	1.00E-02	23.27%	16.27%
	Tlx	1.00E-02	50.61%	41.88%
	NPAS	1.00E-02	89.39%	83.16%
	ZNF317	1.00E-02	14.69%	9.38%

G

Motif	Name	P-value	% of Target Sequences with Motif	% of Background Sequences with Motif
	SpiB	1.00E-04	41.49%	28.87%
	NPAS2	1.00E-03	80.08%	70.46%
	PU.1	1.00E-03	61.00%	50.47%
	IRF8	1.00E-02	46.47%	36.97%
	T1ISRE	1.00E-02	4.15%	1.42%
	HRE	1.00E-02	31.54%	23.64%
	AMYB	1.00E-02	91.70%	85.72%
	NPAS	1.00E-02	88.80%	82.58%
	MITF	1.00E-02	71.37%	63.31%
	PU.1:IRF8	1.00E-02	31.12%	23.75%

H

Motif	Name	P-value	% of Target Sequences with Motif	% of Background Sequences with Motif
	SpiB	1.00E-06	43.02%	28.66%
	STAT4	1.00E-03	83.40%	73.63%
	PU.1	1.00E-03	60.75%	50.44%
	bZIP:IRF	1.00E-03	49.43%	39.59%
	p53	1.00E-02	12.83%	7.58%
	p63	1.00E-02	38.11%	29.70%
	NFkB-p50	1.00E-02	26.79%	19.38%
	Tlx	1.00E-02	47.92%	39.60%
	T1ISRE	1.00E-02	3.77%	1.39%
	IRF1	1.00E-02	23.77%	17.37%

Figure S5. Transcription factor binding site motif analysis reveals the potential TFs controlling commonly up- or downregulated genes in pairs of diseases, related to Figures 2 and 3. The promoter regions (transcription start site ± 2kb) of the top 300 commonly up- or downregulated genes in each pair of diseases from RRHO analysis (Figure 2) were analyzed using the *HOMER* tools. Tables show the sequence motifs, name, *P*-value and frequency (%) in target as well as in background sequences. Top 10 transcription factor binding sites enriched in genes (A) upregulated in T2D and T1D, (B) downregulated in T2D and T1D, (C) upregulated in T2D and AD, (D) downregulated in T2D and AD, (E) upregulated in T2D and MS, (F) upregulated in MS and T1D, (G) upregulated in MS and AD, (H) upregulated in AD and T1D.

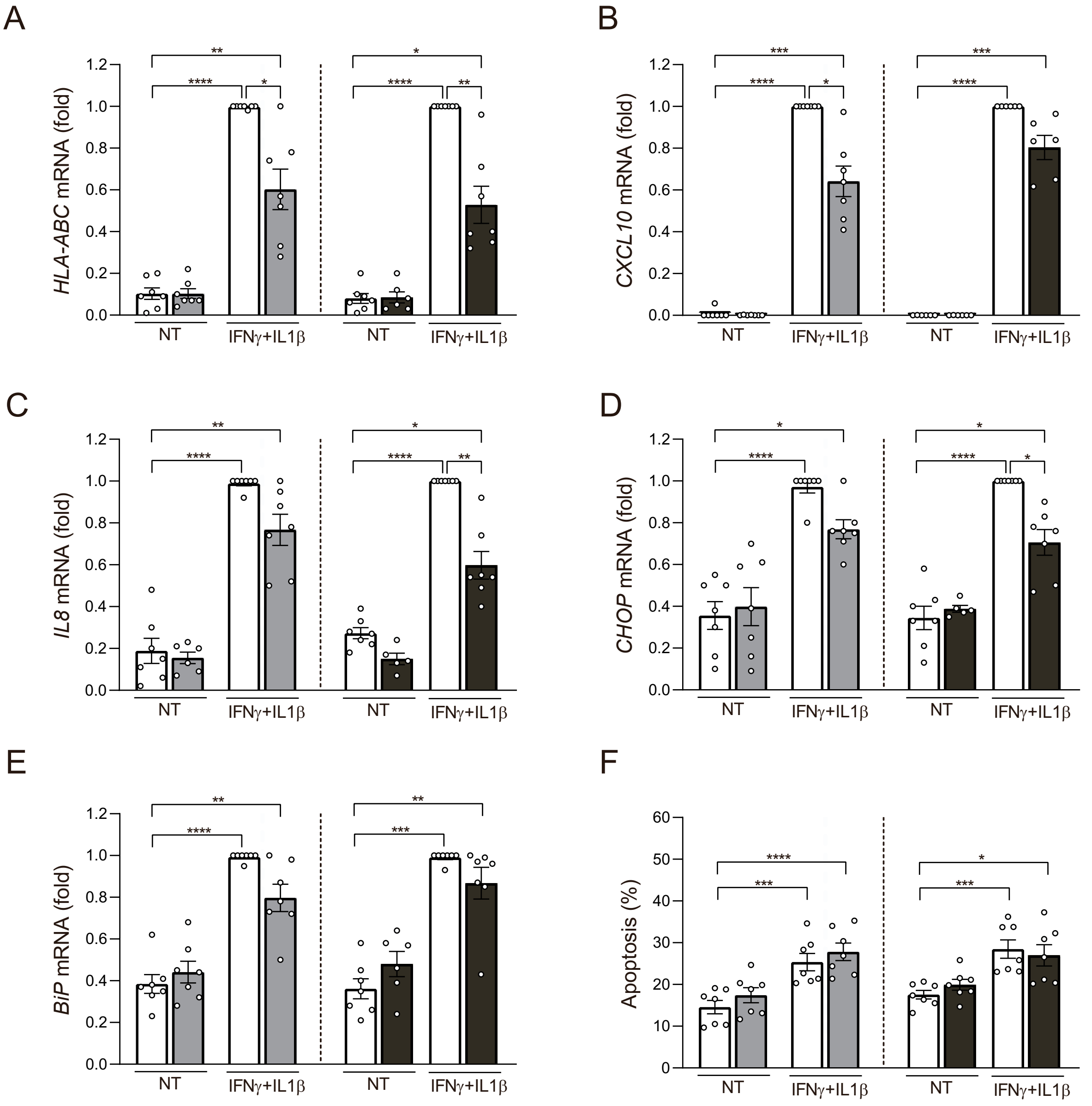

Figure S6. Bromodomain inhibitors attenuate cytokine-induced pro-inflammatory gene expression in EndoC-βH1 cells, related to Figures 4 and 5. EndoC-βH1 cells were pretreated for 6 h with the bromodomain inhibitors iBET-151 (1 µM, grey bars) or GSK046 (1 µM, black bars) and then exposed to IFNγ (1000 U/ml) and IL1β (50 U/ml) or not (non-treated, NT) for 24 h. mRNA expression of *HLA-ABC*, *CXCL10*, *IL-8* and the ER stress markers *CHOP* and *BiP* were analyzed by quantitative real-time PCR. Values were normalized to the geometric mean of the reference genes *β-actin* and *VAPA;* the highest value of each experiment was considered as 1. (F) The percentage of apoptotic cells was counted after 24 h by Hoechst 33342 and propidium iodide staining. Results are mean ± SEM of 5-7 independent experiments. *p<0.05, **p<0.005, ***p<0.001 and ****p<0.0001 by ANOVA followed by Bonferroni correction for multiple comparisons.

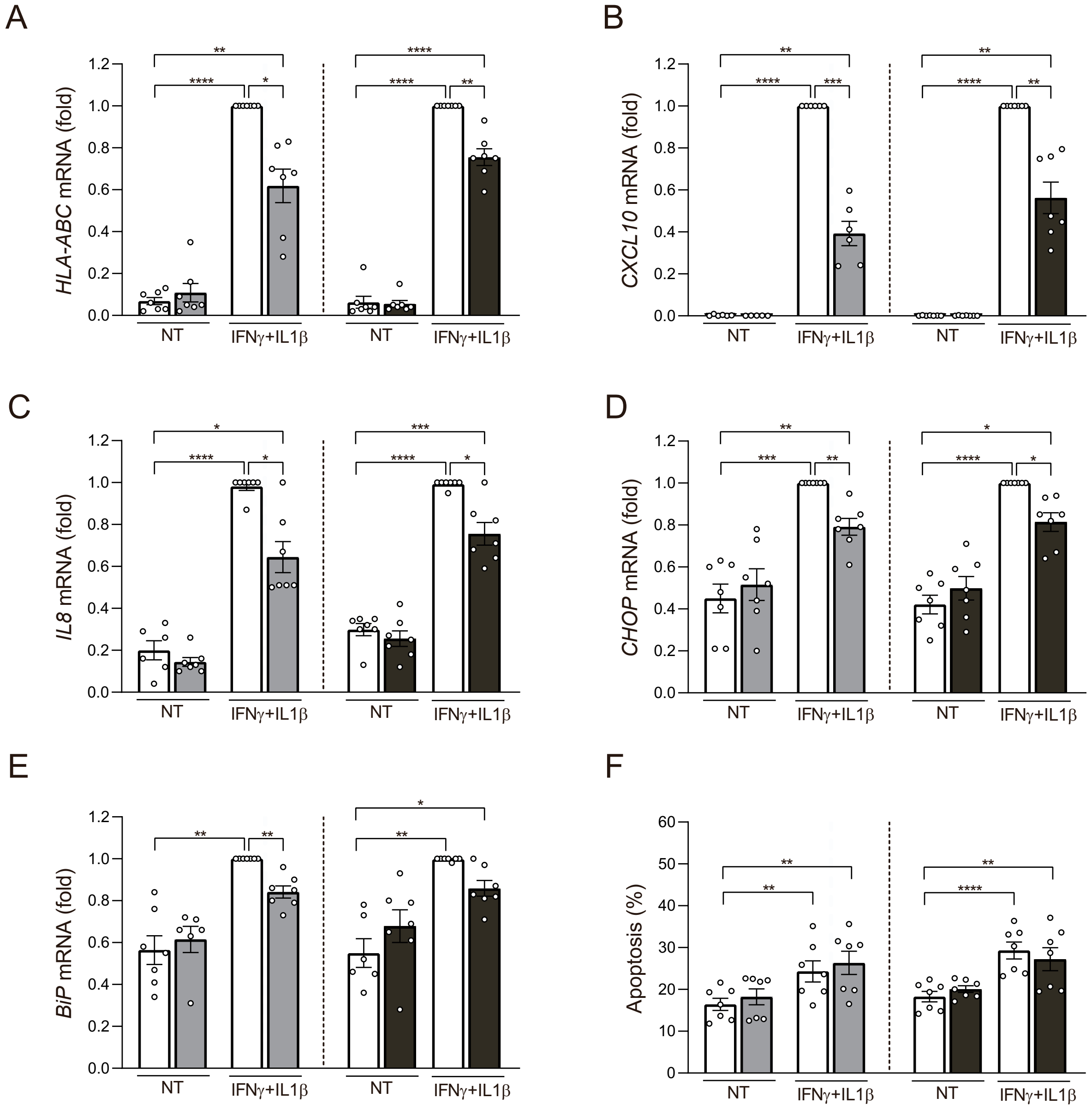

Figure S7. Bromodomain inhibitors attenuate cytokine-induced pro-inflammatory gene expression in EndoC-βH1 cells, related to Figures 4 and 5. EndoC-βH1 cells were pretreated for 6 h with the bromodomain inhibitors iBET-151 (1 µM, grey bars) or GSK046 (1 µM, black bars) and then exposed to IFNγ (1000 U/ml) and IL1β (50 U/ml) or not (non-treated, NT) for 48 h. mRNA expression of *HLA-ABC*, *CXCL10*, *IL-8* and the ER stress markers *CHOP* and *BIP* were analyzed by quantitative real-time PCR. Values were normalized to the geometric mean of the reference genes *β-actin* and *VAPA;* the highest value of each experiment was considered as 1. (F) The percentage of apoptotic cells was counted after 48 h by Hoechst 33342 and propidium iodide staining. Results are mean ± SEM of 5-7 independent experiments. *p<0.05, **p<0.005, ***p<0.001 and ****p<0.0001 by ANOVA followed by Bonferroni correction for multiple comparisons.

Supplementary Tables

Table S1. Expression of leukocyte marker CD45 in the target tissues of four diseases, related to Figure 1.

Disease	Target tissue	Mean TPM in controls	Mean TPM in cases	TPM ratio	$\log_2$ fold change	Adjusted *P*-value
T1D	Pancreatic β-cells	2.6 ± 2.0	15.7 ± 18.1	5.9	1.8	0.069
T2D	Pancreatic islets	1.0 ± 0.6	1.8 ± 1.2	1.7	0.59	0.006
MS	Optic chiasm	9.3 ± 4.2	21.7 ± 20.3	2.3	0.74	0.587
AD	Prefrontal cortex	6.3 ± 3.0	8.1 ± 5.2	1.3	0.36	0.023

CD45 gene expression is displayed as mean ± standard deviation of TPM (Transcript per Million) in cases and controls of the four diseases. Gene expression was quantified using *Salmon 1.4.0*. Mean TPM and TPM ratio were calculated for cases and controls. The $\log_2$ fold change (considering both fold change and estimation for dispersions) and adjusted *P*-value (Benjamini-Hochberg correction) were computed by R package *DESeq2 1.28.1*.

Table S4. Characteristics of human islet donors, related to Figures 5, 6, and 7.

	Age (years)	Gender	BMI (kg/m^2)	Cause of death	β-cell purity (%)
Donor 1	63	Male	21.2	CVD	37
Donor 2	78	Male	25.5	CVD	63
Donor 3	78	Female	26.7	CVD	59
Donor 4	86	Female	22.9	CVD	63
Donor 5	91	Female	22.2	CVD	65
Donor 6	89	Female	23.4	CVD	20
Donor 7	49	Female	31.2	CVD	53
Donor 8	78	Female	25.7	CVD	54

BMI (body mass index); CVD (cardiovascular disease). β-cell purity was assessed by immunostaining for insulin.

Table S5. Sequence of quantitative real-time qPCR primers, related to Figures 5, 7, S6 and S7.

Gene	Primer sequence (5' -> 3')	Direction
ACTB	CTGTACGCCAACACAGTGCT	Forward
	GCTCAGGAGGAGCAATGATC	Reverse
VAPA	TACCGAAACAAGGAAACTAATGGAA	Forward
	GCCTTAAACCTTCATCTCTCAGGT	Reverse
CXCL10	GTGGCATTCAAGGAGTACCTC	Forward
	GCCTTCGATTCTGGATTCAG	Reverse
HLA-ABC	CAGGAGACACGGAATGTGAA	Forward
	TTATCTGGATGGTGTGAGAACC	Reverse
IL-6	AAAAGATGGCTGAAAAAGATGG	Forward
	CTACTCTCAAATCTGTTCTGG	Reverse
IL-8	TGTAAACATGACTTCCAAGCT	Forward
	TTGGAGTATGTCTTTATGCAC	Reverse
CXCL1	AGAACATCCAAAGTGTGAAC	Forward
	TTTCTTAACTATGGGGGATG	Reverse
spliced *XBP1*	CCGCAGCAGGTGCAGG	Forward
	GAGTCAATACCGCCAGAATCCA	Reverse
BIP	Qiagen QuantiTect primer, cat# QT00096404	Forward
		Reverse
CHOP	Qiagen QuantiTect primer, cat# QT00082278	Forward
		Reverse
